# Evaluation of ivabradine plus beta-blocker versus beta-blocker alone in addition to standard care in reducing hospitalization and major adverse cardiovascular event in patients with chronic heart failure: a prospective observational study in tertiary care hospital in central India

**DOI:** 10.1186/s43044-024-00500-7

**Published:** 2024-05-31

**Authors:** Ramyaa Desingu, Shilpa Kaore, Gaurav Kandelwal, S. Balakrishnan

**Affiliations:** 1 Mahatma Gandhi Medical College & Research Institute, Sri Balaji Vidyapeeth, Pondicherry, India; 2 All India Institute of Medical Sciences (AIIMS), Bhopal, India; 3 SAGE Apollo Hospital, Bhopal, India

## Abstract

**Background:**

In recent years, there has been an increase in cases of heart failure, ultimately leading to an increase in hospitalization for heart failure (HF) and cardiovascular mortality. The aim of our study was to evaluate ivabradine combined with beta-blocker versus beta-blocker alone in addition to standard care for chronic heart failure, followed for a period of 6 months for the rate of hospitalization and major adverse cardiovascular event (MACE) in patients with reduced left ventricular ejection fraction (LVEF < 35%).

**Results:**

A total of 64 patients were included in this observational study with 30 patients in the ivabradine + beta-blocker (IVA + BB) group and 34 in the beta-blocker (BB) group. The median (IQR) age of the study sample was 57 (50–62) and 58.5 (55–67) in IVA + BB and BB groups, respectively, with LVEF < 35%. The incidence of the primary endpoint of composite MACE (MI, stroke, death, worsening of HF) was 5 in both groups. The mean heart rate was significantly decreased (*p* < 0.001) at 3-month and 6-month follow-up from baseline in the ivabradine + beta-blocker group as compared to the beta-blocker group alone, while it significantly increased in the beta-blocker group at 3 months (*p* < 0.01) and also at sixth months (*p* < 0.05). Parameters such as the New York Heart Association (NYHA) class and the Minnesota Living with Heart Failure questionnaire (MLWHFQ) were also assessed but did not show significant change.

**Conclusion:**

Overall, observations from the study results show that IVA + BB seems to be overall well tolerated in the study sample, with a somewhat smaller decrease in hospitalization and a delay in MACE events in the sample population enrolled in a tertiary care hospital in India. Further exploration in a larger sample is required concerning the Indian population.

## Background

Heart failure (HF) is a significant health problem in India and worldwide. The annual incidence of new HF events in USA according to Framingham study is 9.2 per 1000 person-years [1]. In India, HF affects 1.3–4.6 million people, with a 0.5–1.8 million yearly incidence [2]. The most common cause of HF is ischemic heart disease (IHD); in recent days, the incidence of IHD has been increasing steadily, which will ultimately lead to a rise in the proportion of patients living with HF and cardiovascular mortality. The incidence of HF mortality within 1 month of discharge from the hospital for acute exacerbation is around 10%, especially patients with heart failure with reduced ejection fraction (HFrEF) have a much shorter life expectancy [3, 4]. In industrialized nations like the USA, there are already over 5.1 million individuals living with heart failure (HF), and by 2030, that figure is predicted to rise to over eight million [5]. To decrease morbidity and mortality, there are various strategies to treat HF, and Ivabradine is one approved for patients mainly to control heart rate. Elevated heart rate has a direct association with major adverse cardiovascular events (MACEs) in patients with HF [6]. Decreasing the heart rate increases the duration of diastole, thereby increasing the oxygen supply and blood flow to the myocardium. Adequate control of heart rate decreases the incidence of MACE and the frequency of hospitalization [7]. Ivabradine is added for patients who had inadequate control of heart rate with a maximum dose of beta-blocker, or who have reached a maximum tolerable dose, or who are intolerant to beta-blocker, to decrease the incidence of both hospitalization and adverse cardiac outcomes [8]. Due to minimal studies on ivabradine in chronic heart failure patients in the Indian population, we have done this prospective, observational study to assess the clinical effectiveness of ivabradine plus beta-blocker versus beta-blocker alone in addition to standard care in reducing hospitalization and major adverse cardiovascular event in patients with chronic heart failure.

## Methods

This prospective observational study was conducted between March 2020 and October 2021 in a tertiary care hospital in India, approved by the institutional Human Ethics Committee vide letter No. IHECPGRMD042 dated March 2020. The study aimed to evaluate the addition of ivabradine plus beta-blocker versus beta-blocker alone in addition to standard care in reducing hospitalization and major adverse cardiovascular events in patients with chronic heart failure in the Indian population. Patients attending All India Institute of Medical Sciences, Bhopal hospital, diagnosed with HF, (1) age ≥ 18yrs, (2) LVEF < 35%, (3) in sinus rhythm, (4) HR ≥ 80bpm (beats per minute) prescribed Ivabradine + Beta-Blocker (IVA + BB) along with standard care were included in the IVA + BB group, and HR ≤ 80bpm who were prescribed BB along with standard care were included in beta-blocker (BB) group and received drugs as prescribed at physician’s discretion. The patients were excluded if (1) acute decompensated heart failure, (2) blood pressure < 90/50mm/Hg, (3) concomitant use of strong CYP3A4 inhibitors like ketoconazole, itraconazole, voriconazole, telithromycin, clarithromycin, nefazodone, ritonavir, saquinavir, nelfinavir, indinavir, atazanavir, grapefruit juice > 1L/day, (4) Sick sinus syndrome, (5) third- and second-degree heart block, (6) concomitant use of calcium channel blocker, (7) severe hepatic and renal impairment as per the FDA recommendations [8].

We followed the patients for 6 months for the primary endpoint: major adverse cardiovascular events (MACE), a combination probability of cardiovascular death, worsening of heart failure, myocardial infarction, and stroke. The secondary objectives were improvement of HR, LVEF, assessment of the quality of life using the Minnesota Living (MLWHFQ) questionnaire, NYHA status, medication adherence, and the study population's SARS CoV-2 infection and vaccination status.

**Follow-up **procedureWe followed the patients at third month and sixth month for:

1. Occurrence of major adverse cardiovascular event (MACE).
2. Occurrence of hospitalization for cardiovascular and non-cardiovascular events.
3. Assessed the quality of life using Minnesota Living with Heart Failure Questionnaire (MLWHFQ) at baseline and at third month, which is a questionnaire consisting of 21 questions, covering various domains of quality of life like, physical domain, emotional domain, and social domain. The total score of this questionnaire is 105, and based on the score the patient’s quality of life was categorized as good, if the score is < 24, moderate if the score is 24 to 45, and poor if the score is > 45 [9, 10].
4. Observed and recorded adverse drug event/adverse drug reaction of drugs prescribed.
5. Data collection regarding COVID-19 infection and vaccination in study population: This datum was collected during their 6th-month follow-up, and the following questions were asked. Regarding previous history of COVID-19 infection patients were asked “Have you suffered from COVID-19 previously?” Further in case vaccination was done, details like the type of vaccine received, whether vaccination completed, any vaccine-related adverse events were also noted. The patients were asked the following questions “Have you taken COVID-19 vaccine?”, if yes, details regarding the type of vaccine, doses taken and adverse event if any after vaccination were collected by asking following questions “Which vaccine you have taken?”, “Have you taken both the doses of vaccine?”, “Did you experience any adverse events following vaccination?”. If the patients have not been vaccinated “What was the reason for not getting vaccinated?” were also asked for.
6. Data collection regarding Medication Adherence: This datum was collected when patients came for 6-month follow-up, and the participants were asked the following questions. They were asked to quantitatively evaluate their adherence using 11 response categories (0, 10, 20,…100%) with the question, “What percent of the time did you take all your Heart failure medication as your doctor prescribed?”. They were also asked to qualitatively rate their adherence on a Likert scale (rating: very poor, poor, fair, good, very good, and excellent). This was done using recall method at two time periods, one week and one month. We also collected data regarding the barriers to maintain medication adherence by asking “What was the reason to skip medications in between?” All the adherence information were captured for the drugs prescribed to our study population [11].

### Statistical analysis

Data were analyzed using statistical software packages SPSS version 26.0 and Stata version 12B. Categorical variables such as clinical presentation were tabulated using frequencies and proportions. It was analyzed and compared using either the Chi-square test or Fisher’s exact test, as needed. Continuous data were analyzed using mean ± standard deviation or median and interquartile range and then compared using paired t-test or independent t-test depending on the distribution type of the variable under analysis. Two-tailed statistical tests with a significance level of 0.05 were considered. The Cox proportion hazard model and the survival curves were analyzed using Nelson–Aalen cumulative hazard estimates to compare our study's primary endpoint.

## Results

In our study, a total of 64 patients were enrolled and followed up for 6 months for the occurrence of the primary objective, hospitalization for cardiovascular and non-cardiovascular events, and MACE and other secondary outcomes. The median (IQR) age of patients in the study groups was 57 (50–62) and 58.5 (55–67) in the IVA + BB group and BB group, respectively, depicting that the patients in both groups were similar in age. The mean age of patients included in our study was lower than compared to other studies, such as the SHIFT & INTENSIFY trial, while the gender distribution was comparable.

The baseline characteristics of the patients in the study groups along with the data of other concomitant medications prescribed as a part of standard care as ACE inhibitors, ARBs, ARNI, spironolactone, torsemide, dapagliflozin, statins, aspirin, clopidogrel, and antianginal drugs; their proportions were compared between the groups are shown in Table 1. Beta-blockers prescribed in our study were metoprolol succinate and carvedilol; metoprolol was prescribed in majority of the patients, 80% in the IVA + BB group and 73.5% in the BB group. The baseline LVEF values were expressed in mean ± SD as 25.83 ± 5.58 for IVA + BB and 28.38 ± 6.36 for the BB group. Overall, at baseline, the study groups were comparable (Table 1).

**Table 1:** Baseline characteristics of patients in both the study groups

Characteristics		IVA + BB (*n* = 30)	BB (*n* = 34)	*P*-value
Age—median (IQR)		57 (50–62)	58.5 (55–67)	0.79
Gender n (%)	Male	24 (80)	31 (91.2)	0.17
	Female	6 (20)	3 (8.8)	
Disease duration—median (IQR)		30 (42)	24 (30)	0.09
Concomitant disease n (%)	Diabetes	14 (46.7)	10 (29.4)	0.19
	Hypertension	10 (33.3)	7 (20.6)	0.27
	Thyroid disorder	4 (13.3)	2 (5.9)	0.40
Smoking—n (%)		19 (63.3)	16 (47.1)	0.21
Disease etiology—n (%)	Ischemic	20 (66.7)	18 (52.9)	0.31
	Non-ischemic	10 (33.3)	16 (47.1)	
LVEF- Mean ± SD		25.83 ± 5.58	28.38 ± 6.36	0.09
NYHA class (Baseline) n (%)	I	2 (6.7)	6 (17.6)	0.51
	II	15 (50)	17 (50)	
	III	12 (40)	9 (26.5)	
	IV	1 (3.3)	2 (5.9)	
Concomitant medication details n (%)	ACE inhibitor	10 (33.3)	13 (56.5)	0.79
	ARB	2 (6.7)	1 (2.9)	0.59
	ARNI	5 (16.7)	8 (23.5)	0.54
	Spirinolactone	27 (90.0)	28 (82.4)	0.48
	Torsemide	29 (96.7)	29 (85.3)	0.20
	Dapaglifozin	3 (10)	0	0.09
	Statins	26 (44.1)	33 (55.9)	0.31
	Metoprolol	24 (80)	25 (73.5)	0.57
	Carvedilol	6 (20)	9 (26.5)	
Minnesota Living with Heart Failure Questionnaire:				
Total score—median (IQR)		36 (22)	36 (26.25)	0.77
Physical domain score (PDS)		13 (14.75)	16 (16.25)	0.85
Emotional domain score (EDS)		6 (5.75)	10 (7.5)	0.60
Social domain score (SDS)		7 (6.25)	6 (5.25)	0.91
Minnesota Living with Heart Failure Questionnaire—Category				
Good (< 24)		5 (16.7)	7 (20.6)	0.66
Moderate (24–45)		17 (56.7)	16 (47.1)	
Poor (> 45)		7 (23.3)	11 (32.4)	

BMI, Body Mass Index; IQR, Interquartile Range; CAD, Coronary Artery Disease; LVEF, Left Ventricular Ejection Fraction; CCB, Calcium Channel Blocker; ACE, Angiotensin-Converting Enzyme; ARB, Angiotensin Receptor Blocker; SD, Standard Deviation; NYHA, New York Heart Association

The primary and secondary endpoint of the study was analyzed using Cox proportion hazard model, and the survival curves was analyzed using Nelson–Aalen cumulative hazard estimates.

The primary endpoint in our study was MACE: a composite of MI, stroke, worsening of HF and cardiovascular death, which occurred in 5 of 30 (16.7%) patients in the IVA + BB group and 5 of 34 (14.7) in the BB group (hazard ratio (HR), 1.13 (95%CI, 0.32 to 3.19); *p* = 0.84)), and the difference was not statistically significant (Table 2). The incidence of all-cause hospitalization in the IVA + BB group was 7 (23.3%) and 8 (23.5) in the BB group (hazard ratio (HR), 0.99 (95% CI, 0.36 to 2.73); *p* = 0.98)). One of the components in MACE was cardiovascular-related death, which occurred in 1 patient in each group (hazard ratio (HR), 1.1 (95% CI, 0.07 to 18.1); *p* = 0.92) [Table 2].

**Table 2:** NYHA class at baseline and at the end of study within the study groups

	Class	At baseline- *n* (%)	At end of study- *n* (%)	*P*-value
Ivabradine + beta-blocker	I	2 (6.7)	4 (13.8)	0.72
	II	15 (50)	13 (44.8)	
	III	12 (40)	10 (34.5)	
	IV	1 (3.3)	2 (6.9)	
Beta-blocker	I	6 (17.6)	7 (21.2)	0.97
	II	17 (50)	15(45.5)	
	III	9 (26.5)	9 (23.3)	
	IV	2 (5.9)	2 (6.1)	

**Fig. 1:**
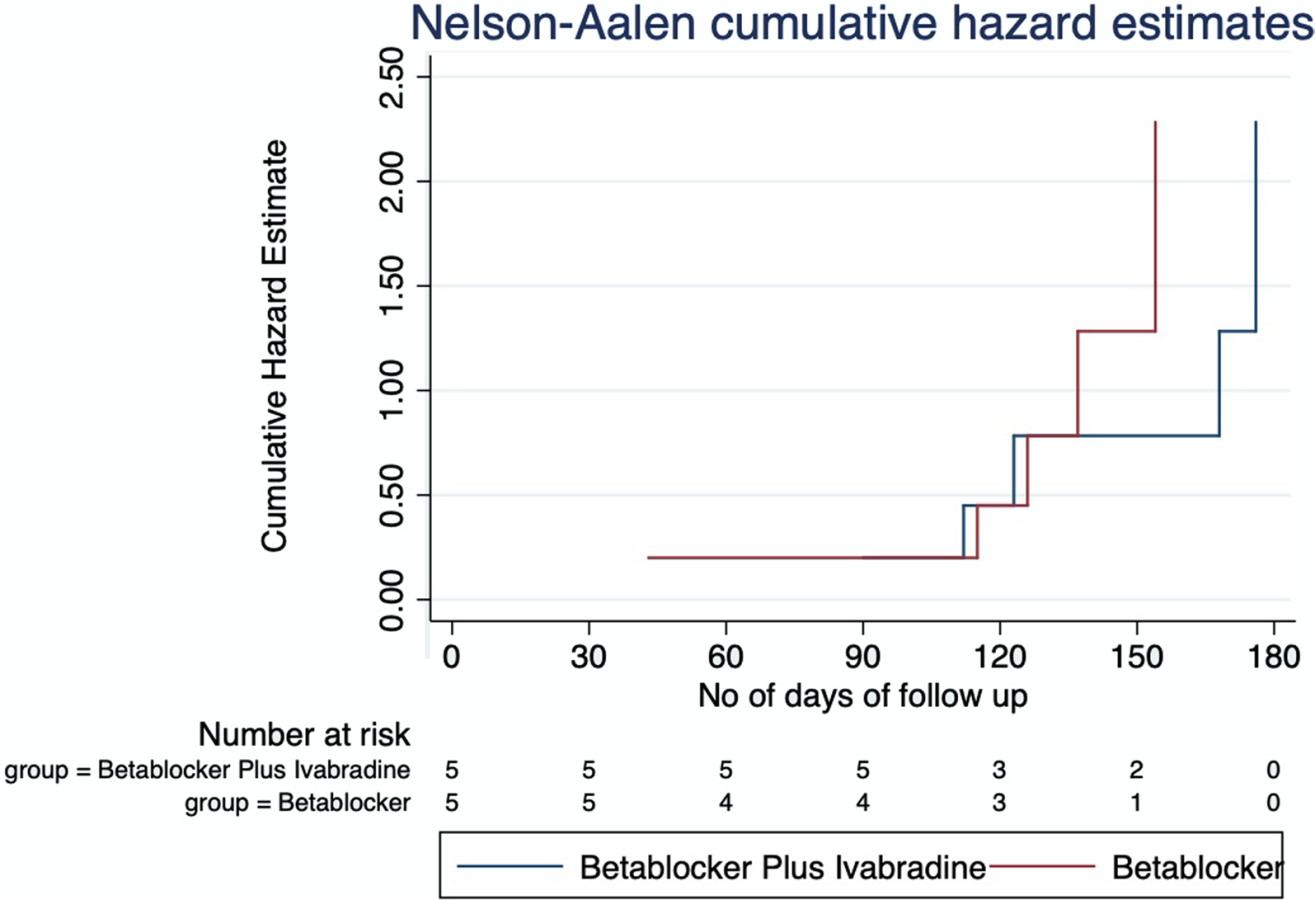
Cumulative hazard estimate of MACE in the study groups

**Fig. 2:**
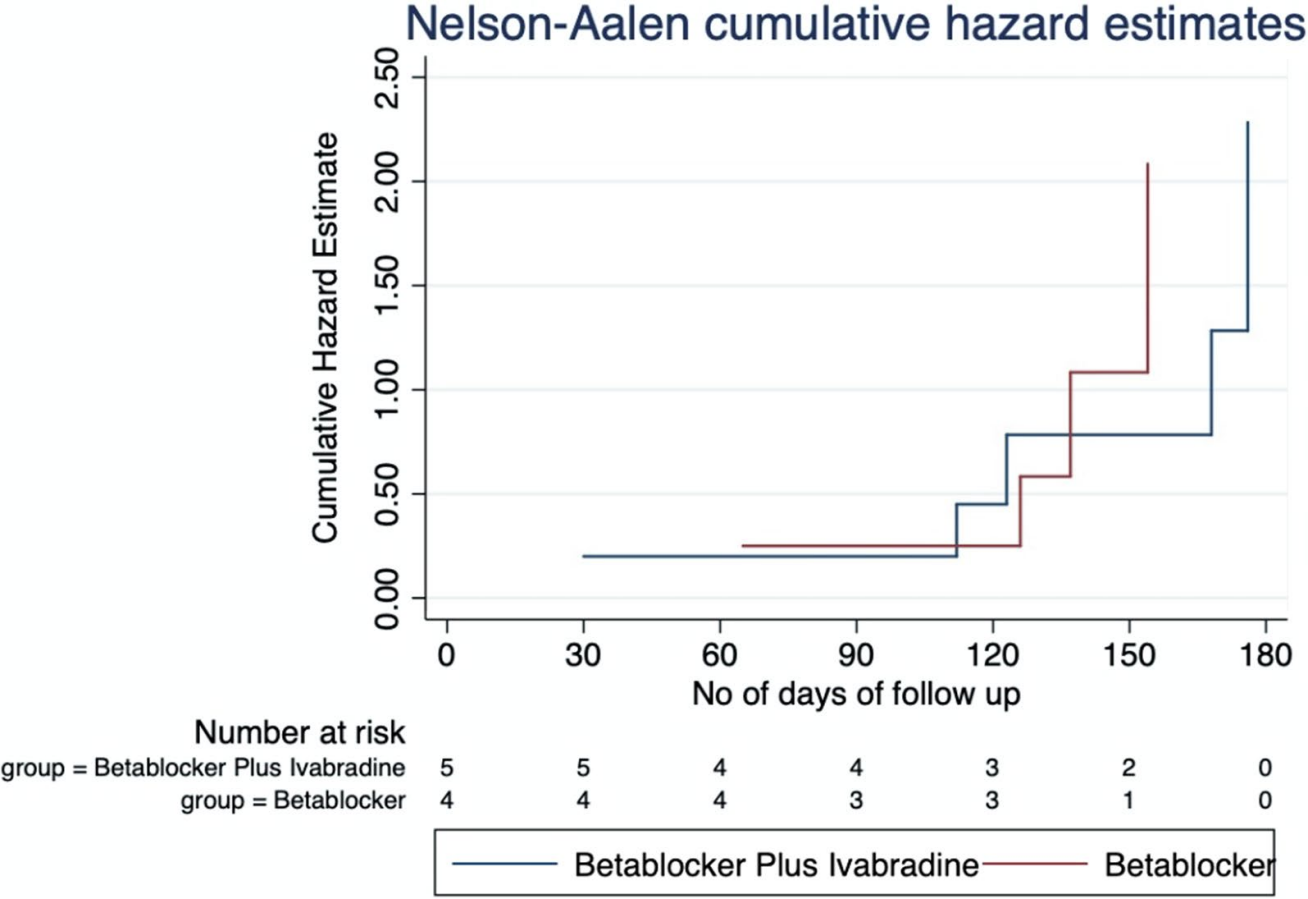
Cumulative hazard estimate of all-cause hospitalization in the study groups

Figures 1 and 2 show the cumulative hazard for the study groups using Nelson–Aalen estimator. This survival estimator is used, since it performs better when the sample size is small; hence, we have used it to depict the survival end points [12, 13]. For the primary endpoint MACE, the hazard estimates increased over a period of time for both the groups. Ivabradine + beta-blocker group attained hazard risk of 2.25 in 180 days and beta-blocker group attained the same hazard risk at 150 days (Fig. 1). The secondary end point, like all-cause hospitalization, the hazard estimates increased over a period of time, and the hazard risk of 2.25 was attained at 150 days for beta-blocker group and 180 days for ivabradine + beta-blocker group (Fig. 2).

Monitoring was done throughout the study regarding the adverse drug reactions, which indicates that the drugs were well tolerated, and no significant adverse drug reaction was noted during the study.

The incidence of all-cause hospitalization, hospitalization for HF, and hospitalization for a cardiac cause other than HF, like hospitalization for angiography, angioplasty, and coronary bypass graft surgery, were also compared similarly to the primary endpoints.

**Fig. 3:**
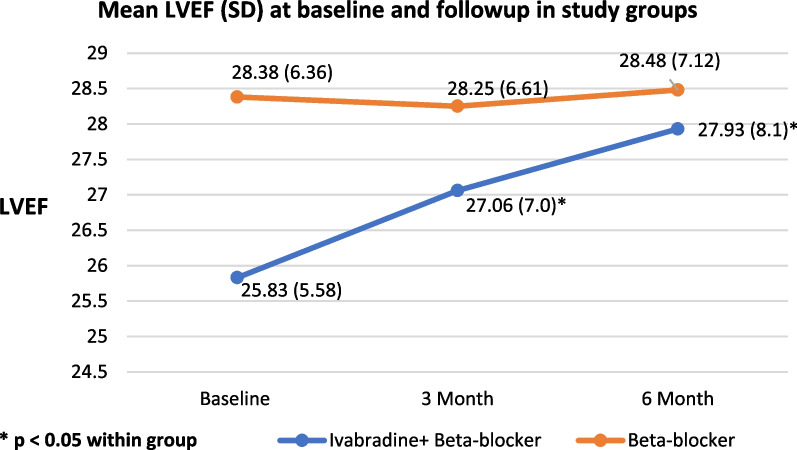
LVEF of both study groups at baseline to the end of the study as mean + SD

**Fig. 4:**
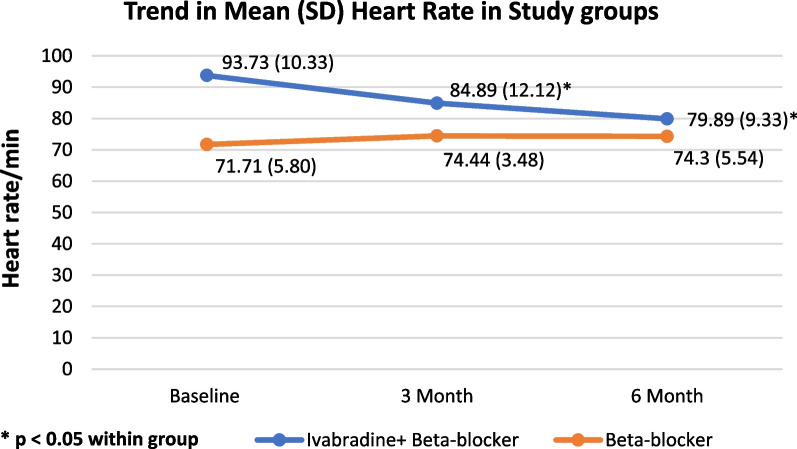
Mean heart rate during treatment in both the study groups from baseline to end of study

There was a statistically significant improvement in LVEF from 25.83 ± 5.58 to 27.93 ± 8.1 in the IVA + BB group [Fig. 3]. The mean HR reduced significantly in the IVA + BB group from 93.73 ± 10.33 to 79.89 ± 9.33 (*p* < 0.001), whereas in the BB group, HR increased from 71.71 ± 5.80 to 74.30 ± 5.54 (p < 0.05) [Fig. 4]. Regarding the quality of life using MLWHF questionnaire in the IVA + BB group, there was an increase in the proportion of patients in good category (score < 24) 27.6% in IVA + BB group and 23.5% in BB group, but this was not significant. The medication adherence percentage was more than 90% in both study groups.

## Discussion

The FDA approved Ivabradine to reduce the rate of hospitalization and cardiovascular-related death in patients with HFrEF, who are in sinus rhythm with resting heart rate ≥ 70 beats per minute and either is on maximally tolerated doses of beta-blockers or have a contraindication to beta-blocker use [8].

In our study, a total of 64 patients were enrolled and followed up for 6 months for the occurrence of primary objective MACE and for other secondary outcomes. The median (IQR) age of patients in the study groups was 57 (50–62) and 58.50 (55–67) in the IVA + BB group and BB group, respectively, depicting that the patients in both groups were similar for age. The mean age of patients included in our study was lower than compared to other studies, such as SHIFT & INTENSIFY trial, while the gender distribution was comparable.

In the SHIFT trial, the mean age of patients in the ivabradine group was 60.7 ± 11.2 and 60.1 ± 11.5 in the placebo group, and in the INTENSIFY study, the mean age was 67 ± 11.7, and this further supports our result that a similar age group patients were included in our study. The gender-wise distribution showed that most patients were male, 80% in the IVA + BB group and 91.2% in the BB group; this was similar to gender observed in other major trials like the SHIFT and ETHIC-AHF study. In the SHIFT trial, 76% of patients were male, and in the ETHIC-AHF study, 71.9% were male [14, 15].

The analysis of the primary composite endpoint MACE in our study showed no significant difference between the study groups. Even though the hazard ratio for MACE was 1.13, indicating that there were increased events in the IVA + BB group, and the heart rate is one of the variables directly correlated with the number of adverse cardiac events [16]. In our study, HR was one of the inclusion criteria; patients with HR (35) ≥ 80bpm were included in the IVA + BB group, and patients with HR < 80 bpm were included in the BB group and were followed for six months for the occurrence of primary and secondary events. Since it is an observational study, and due to ethical reasons, we could not make the groups comparable concerning HR. Hence, the baseline mean heart rate in the IVA + BB group was 93.73 ± 10.33, and 71.71 ± 5.80 in the BB group. There was a 22bpm difference in HR between the study groups at baseline. In the SHIFT study, Bohm et al., in 2010, studied the association between HR and adverse cardiovascular outcomes, in which they found that for every beat increase in HR, there was a 3% increase in the risk of the primary composite endpoint (cardiovascular death and hospitalization for HF) [16]. Upon extrapolating, the patients in the IVA + BB group had a 66% increased risk of occurrence of events at baseline; despite that, the number of MACE events in the IVA + BB group was similar (5-IVA + BB; 5-BB), indicating that ivabradine in addition to standard care decreased the occurrence of adverse cardiac outcomes. This can be further supported by another study, a meta-analysis done by Zhang D. et al. in 2016, to observe the effect of higher resting heart rate on all-cause and cardiovascular mortality in the general population [17]. The authors have quoted that for every 10-beat increment in resting heart rate, the risk of all-cause and cardiovascular mortality increased by 9% and 8%, respectively. Also, a much more significant increase in risk of cardiovascular mortality was observed in patients with HR > 90 bpm [17].

Regarding the dose of ivabradine in our study, the initial starting dose was 5mg, and most patients in the combination group were maintained in this dose. Only in one patient was the dose increased to 7.5mg due to inadequate control of HR. The average reduction of HR at 6 months in the ivabradine + beta-blocker group was 13.84 bpm, which was a significant difference when compared to the baseline HR (*p* = 0.001). The results observed in our study were parallel to those observed in the SHIFT and INTENSIFY studies. The study drug ivabradine was well tolerated in our study population; only two patients have stopped ivabradine due to bradycardia but were asymptomatic and found during regular visits to the outpatient department.

In the SHIFT study, treatment with ivabradine caused an average reduction of 15 bpm from a baseline value of 80 bpm. In the INTENSIFY study, there was a 15.4 bpm reduction in HR from a baseline value of 79.9 bpm [18]. Further, therapy with ivabradine should be discontinued if the heart rate is < 50bpm at a dose of 2.5mg twice daily. In SHIFT trial, 1% of patients had a specific adverse effect called phosphenes that are transient brightness in the visual field triggered by exposure to bright light. In our study, this adverse effect was not reported in patients prescribed ivabradine. Since our sample size was small, further studies with larger sample sizes may light up the incidence of this adverse effect in the Indian population.

In our study, the maximum tolerated dose for beta-blocker was 100mg daily, and most of the patients in both groups attained less than or equal to 50% of the target dose. In SHIFT study also, only 26% of patients reached the target dose in both the ivabradine and placebo groups [4].

In our study, we observed that the LVEF% is not directly related to the symptomatology; few patients with LVEF < 15% were in the NYHA class II category, and in few patients, even with LVEF of 30%, the NYHA grade was IV, although LVEF is useful, in classifying patients as HFrEF or HFpEF and for the management of HF. We also categorized patients according to LVEF as > 35%, 25–35%, and < 25%. Most patients had LVEF 25–35%, 66.7% in the IVA + BB group, and 76.5% in the BB group, which was insignificant. During follow-up, 10.3% of patients in the IVA + BB group and 6.1% in the BB group had improvement in LVEF (> 35%), but this improvement was insignificant. One patient in the IVA + BB group and one patient in the BB group had undergone coronary artery bypass graft surgery and angioplasty and had improvement in LVEF (> 35%). In the INTENSIFY study, the proportion of patients with LVEF < 35% decreased from 26.6% at baseline to 17.4% at 4 months, but the authors did not mention whether this result was significant. However, the authors claimed an improvement in LVEF; this could be attributed to the higher number of patients included in their study (*n* = 1941) compared to our study (*n* = 64). (16).

The quality of life in our study was assessed using Minnesota Living with Heart Failure Questionnaire. This was done in two time points at baseline and at three months in both the study groups; the score correlated with poorer response. At baseline, the total scores, physical domain score (PDS) (Item No-2,3,4,5,6,7,12,13), emotional domain score (EDS) (Item No-17,18, 19, 20, 21), and social domain score (SDS) (Item No-8, 9, 10, 14, 15, 16) between the groups were similar and did not show any significant difference. During follow-up at 3 months, the total scores, PDS, EDS and EDS showed no significant improvement in the IVA + BB or BB group. However, the total score was somewhat decreased, from 36 to 28 in IVA + BB, but not in the BB group (Tables 3 and 4).

**Table 3:** Comparison of Minnesota Living With Heart Failure Questionnaire (MLWHF) at baseline

BASELINE- Score – median (IQR)	Ivabradine + Beta-blocker	Beta-blocker	*P*-value
Total score	36 (22)	36 (26.25)	0.77
Physical domain score (PDS)	13 (14.75)	16 (16.25)	0.85
Emotional domain score (EDS)	6 (5.75)	10 (7.5)	0.60
Social domain score (SDS)	7 (6.25)	6 (5.25)	0.91

**Table 4:** Comparison of Minnesota Living With Heart Failure Questionnaire (MLWHFQ scores at third month

3-month score—median (IQR)	Ivabradine + Beta-blocker	Beta-blocker	*P*-value
Total score	28 (21.5)	35 (25.5)	0.30
Physical domain score (PDS)	16 (13)	16 (15.5)	0.80
Emotional domain score (EDS)	5 (5)	10 (8.5)	0.12
Social domain score (SDS)	5 (6)	6 (6.5)	0.77

The most common reason told by the patients for nonadherence is cost of drug in both the groups. Regarding the COVID-19 vaccination, we have found that increased proportion of patients, that is 62.1% in ivabradine + beta-blocker group and 51.5% in beta-blocker group, were not vaccinated for COVID-19. The common reasons quoted by the patients were hesitancy due to patient’s co-morbid condition and fear of adverse drug reactions due to COVID-19 vaccine. Compared to ivabradine + beta-blocker group, the vaccination rate in beta-blocker group was higher, but it was not significant.

## Conclusion

Overall, the result of our study shows that ivabradine was well tolerated in the study population, and it effectively and significantly decreased HR. Since HR is directly correlated with overall outcomes, a significant decrease in HR is associated with a decrease in cardiovascular outcomes like cardiovascular death and hospitalization for the worsening of HF. Another important finding was a statistically significant improvement in LVEF in the IVA + BB group, despite surgical intervention and the background medication, which were similar in both groups. However, no statistically significant difference regarding parameters like NYHA class and MLWHF score was found. Regarding the NYHA class, a shift of patients to NYHA class I was observed, indicating a favorable outcome in the IVA + BB group. Also, the MLWHF score was decreased in the IVA + BB group, suggesting a better quality of life in terms of physical, social, and emotional domains.

## Limitation

Apart from the expected selection and observer bias inherent in an observational study design in our study, the other limitations were lesser sample size (due to coincidence with SARS CoV-2 era with lockdown, state of panic, and hesitancy to move out), the disparity in the baseline HR (since it was not a RCT), and short-term follow-up for mortality and morbidity outcomes. In the future, more patients may be followed up for a longer duration of time, which might reveal the real benefits of ivabradine in patients with HF in the therapeutic scenario.

## Data Availability

Yes, we have the availability of data for the study. Data were collected for this observational study as per IHEC-approved protocol. Data will be made available on reasonable request.

## Abbreviations

ARNI: Angiotensin receptor/ neprilysin inhibitor
BB: Beta-blocker
bpm: Beats per minute
CAD: Coronary artery disease
CHF: Congestive heart failure
CVS: Cardiovascular
EDS: Emotional domain score
EF: Ejection fraction
ETHIC-AHF: Early Therapy with Ivabradine in patients with Congestive Acute Heart Failure
HF: Heart failure
HFpEF: Heart failure with preserved ejection fraction
HFrEF: Heart failure with reduced ejection fraction
HR: Heart rate
INTENSIFY: PractIcal daily effectiveNess and TolEraNce of ivabradine in chronic Systolic heart
IQR: Interquartile range
IVA: Ivabradine
LVD: Left ventricular dysfunction
LVEF: Left ventricular ejection fraction
MACE: Major adverse cardiovascular event
MI: Myocardial infarction
MLWHF: Minnesota Living with Heart Failure questionnaire
NYHA: New York Heart Association
PDS: Physical domain score
RCT: Randomized controlled trial
SARS Co-V-2: Severe acute respiratory syndrome coronavirus 2
SDS: Social domain score
SHIFT: Systolic Heart Failure Treatment With the IF Inhibitor Ivabradine Trial
SPSS: Statistical Package for the Social Sciences
